# Multidimensional, quantitative assessment of PD-1/PD-L1 expression in patients with Merkel cell carcinoma and association with response to pembrolizumab

**DOI:** 10.1186/s40425-018-0404-0

**Published:** 2018-10-01

**Authors:** Nicolas A. Giraldo, Peter Nguyen, Elizabeth L. Engle, Genevieve J. Kaunitz, Tricia R. Cottrell, Sneha Berry, Benjamin Green, Abha Soni, Jonathan D. Cuda, Julie E. Stein, Joel C. Sunshine, Farah Succaria, Haiying Xu, Aleksandra Ogurtsova, Ludmila Danilova, Candice D. Church, Natalie J. Miller, Steve Fling, Lisa Lundgren, Nirasha Ramchurren, Jennifer H. Yearley, Evan J. Lipson, Mac Cheever, Robert A. Anders, Paul T. Nghiem, Suzanne L. Topalian, Janis M. Taube

**Affiliations:** 1 Department of Dermatology, Johns Hopkins University School of Medicine, Baltimore, MD USA; 2 Department of Pathology, Johns Hopkins University School of Medicine, Baltimore, MD USA; 3 Department of Oncology, Johns Hopkins University School of Medicine, The Sidney Kimmel Comprehensive Cancer Center, Baltimore, MD USA; 4 Department of Biostatistics, Johns Hopkins University School of Medicine, Baltimore, MD USA; 5 Department of Surgery, Johns Hopkins University School of Medicine, Baltimore, MD USA; 6 Division of Dermatology, Department of Medicine, University of Washington Medical Center, Seattle, WA USA; 7 Cancer Immunotherapy Trials Network, Fred Hutchinson Cancer Research Center, Seattle, WA USA; 8 Merck Research Laboratories, Kenilworth, NJ USA; 9 The Bloomberg~Kimmel Institute for Cancer Immunotherapy, Baltimore, MD USA

**Keywords:** PD-1, PD-L1, Merkel cell, Multispectral immunofluorescence, Pembrolizumab

## Abstract

**Background:**

We recently reported a 56% objective response rate in patients with advanced Merkel cell carcinoma (MCC) receiving pembrolizumab. However, a biomarker predicting clinical response was not identified.

**Methods:**

Pretreatment FFPE tumor specimens (*n* = 26) were stained for CD8, PD-L1, and PD-1 by immunohistochemistry/immunofluorescence (IHC/IF), and the density and distribution of positive cells was quantified to determine the associations with anti-PD-1 response. Multiplex IF was used to test a separate cohort of MCC archival specimens (*n* = 16), to identify cell types expressing PD-1.

**Results:**

Tumors from patients who responded to anti-PD-1 showed higher densities of PD-1+ and PD-L1+ cells when compared to non-responders (median cells/mm^2^, 70.7 vs. 6.7, *p* = 0.03; and 855.4 vs. 245.0, *p* = 0.02, respectively). There was no significant association of CD8+ cell density with clinical response. Quantification of PD-1+ cells located within 20 μm of a PD-L1+ cell showed that PD-1/PD-L1 proximity was associated with clinical response (*p* = 0.03), but CD8/PD-L1 proximity was not. CD4+ and CD8+ cells in the TME expressed similar amounts of PD-1.

**Conclusions:**

While the binomial presence or absence of PD-L1 expression in the TME was not sufficient to predict response to anti-PD-1 in patients with MCC, we show that quantitative assessments of PD-1+ and PD-L1+ cell densities as well as the geographic interactions between these two cell populations correlate with clinical response. Cell types expressing PD-1 in the TME include CD8+ T-cells, CD4+ T-cells, T_regs_, and CD20+ B-cells, supporting the notion that multiple cell types may potentiate tumor regression following PD-1 blockade.

**Electronic supplementary material:**

The online version of this article (10.1186/s40425-018-0404-0) contains supplementary material, which is available to authorized users.

## Background

Merkel cell carcinoma (MCC) is an aggressive uncommon cutaneous malignancy, for which two main etiologies have been described: Merkel cell polyomavirus (MCPyV) infection, associated with approximately 80% of cases; and ultraviolet light exposure, which accounts for the remaining 20% [1]. Patients with MCC often exhibit oligoclonal lymphocyte-mediated and antibody-mediated immunity against MCC tumor antigens [2–4], with complex arrangements of immune cells, including B cells, CD4+ and CD8+ T cells, macrophages and regulatory T cells [5]. CD8+ tumor-infiltrating lymphocytes (TIL) [4, 6] and tumor cell PD-L1 expression [7] have been associated with improved patient survival, indicating that the immune system is able to exert some control over this aggressive neoplasm.

PD-1 is an inhibitory receptor expressed on various immune cell subsets, including CD8+ and CD4+ T cells, B cells, and natural killer cells [8]. The interaction between PD-1 and its ligands downregulates immune cell activation, proliferation, survival and cytokine production [8, 9]. For this reason, therapeutic blockade of the PD-1/PD-L1 checkpoint has been embraced as a strategy to enhance antitumor immunity, with durable efficacy in some patients with multiple tumor types [10]. We recently reported that patients with advanced MCC receiving first-line treatment with pembrolizumab (anti-PD-1) experienced an objective response rate of 56% [11]. Also, patients treated with avelumab (anti-PD-L1) showed a 66% and 32% response rate when received in the first and second/third line settings, [12, 13] respectively. Many efforts to discover and validate biomarkers of response to anti-PD-(L)1 are currently underway. The best-studied biomarker is tumor PD-L1 protein expression, measured by immunohistochemistry (IHC) and graded by a pathologist as either “positive” or “negative”. Across multiple solid tumor types, it has been shown that patients whose pre-treatment tumors are PD-L1+ demonstrate an enriched objective response rate to anti-PD-(L)1, compared to their PD-L1- counterparts [14]. However, we found that the simple presence or absence of tumor cell PD-L1 expression in MCC did not correlate with anti-PD-1 response [11]. In the current study, we expanded our histopathologic analysis of the MCC TME using next-generation digital pathology-assisted quantitative methods, including topographic quantitative density analyses and spatial proximity analyses, to assess the density, distribution, and proximity of CD8+, PD-1+ and PD-L1+ cell populations. We found that the density of PD-1+ cells or PD-L1+ cells, and the number of PD-1+ cells in close proximity to PD-L1+ cells, each correlated with clinical response. While it is assumed that PD-1 is mostly involved in regulation of CD8+ T-cell activity in the TME, we discerned multiple immune cell subsets which may contribute to PD-1 biomarker relevance.

## Methods

### Case selection

This study was approved by the institutional review board of each participating institution. All participants provided written informed consent. Twenty-six patients with stage IIIB or IV MCC were enrolled from January 2015 until December 2015 and received at least one dose of pembrolizumab on a phase 2, single-cohort, multicenter clinical trial [11]. Objective responses were assessed by the investigators according to RECIST, version 1.1 [15]. A patient was considered to have an objective response to therapy if they demonstrated either a complete response (CR) or partial response (PR), per data analysis on 08/01/2016. A single representative pre-treatment formalin-fixed paraffin-embedded (FFPE) tumor specimen was chosen from each patient for additional studies. The minimum criteria for inclusion was a contiguous viable tumor deposit measuring >1 mm^2^ in size.

A second cohort of 16 FFPE specimens acquired between 11/2002–04/2011 from 16 unique patients with stage IA- IV MCC was obtained from the Johns Hopkins Hospital (JHH) surgical pathology archives [7]. This cohort did not receive anti-PD-1 therapy. H&E slides from each case were reviewed by a board-certified dermatopathologist to confirm the diagnosis. A single representative FFPE tumor block was chosen for additional studies.

### Single IHC or IF stains

Serial 4 μm FFPE pre-treatment tumor specimens from patients receiving anti-PD-1 were stained for CD8 (*n* = 23/26, mAb clone 144B, Dako, Carpinteria, CA), PD-1 (*n* = 16/26, goat polyclonal Ab, R&D Systems, Minneapolis, MN) or PD-L1 (*n* = 25/26, mAb clone 22C3, Merck Research Laboratories, White House Station, NJ) by IHC/IF, as previously described [11]. Archival specimens from patients not treated with anti-PD-1 were stained for NKp46 using IHC. Appropriate positive and negative controls for each marker were run with every batch.

### Pathologist interpretation of PD-L1 IHC

PD-L1 expression on tumor cells (TC) and immune cells (IC) was scored by two pathologists blinded to patient outcomes (AS, JC). PD-L1 expression was assessed as none (< 1%), 1%, 2–4%, 5–9%, 10–19%, and at increasing 10% intervals. PD-L1+ TC or IC had ≥1% positive cells.

### Digital image analysis for cell densities and proximity analysis using single IHC/IF stains

**Fig. 1:**
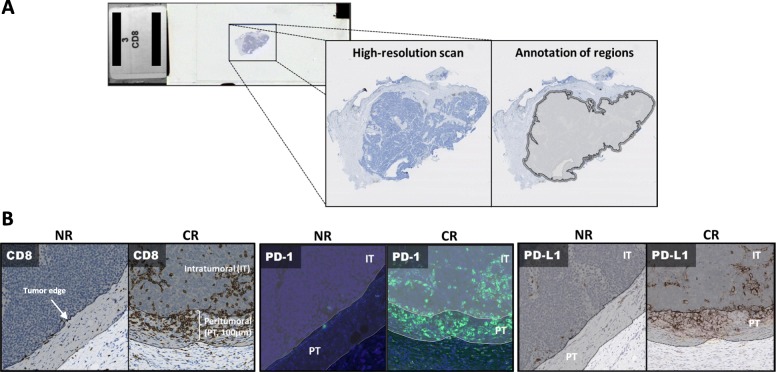
Tumor regions were annotated on high-resolution digital scans of slides stained by IHC/IF. **a** Peritumoral (PT, 100 um) and intratumoral (IT) regions were annotated. **b** Representative images for CD8 (brown), PD-1 (green) and PD-L1 (brown) staining from a non-responder (NR) and complete responder (CR)

Slides stained for PD-L1, PD-1 or CD8 were scanned using Aperio ScanScope (Leica Biosystems Imaging, Buffalo, IL). NKp46-stained slides were scanned using a NanoZoomer XR (Hamamatsu Photonics, Hamamatsu City, Japan). The resultant slide images were assessed using digital image analysis software (HALO V2.0, Indica Labs, Corrales, NM). The tumor border was annotated by a pathologist, and the area encompassed by this region was designated as intratumoral (IT). A 100 μm distance beyond the tumor-stroma interface was designated as the peritumoral (PT) region, Fig. 1. The number of positive cells per mm^2^ displaying CD8, PD-1, PD-L1 or NKp46 was assessed as a continuous variable in the IT, PT or total (PT and IT) TME regions. PD-L1 expression was quantified in the total fraction of tissue surface area (total pixels positive/total pixels). Acellular and necrotic areas were excluded from analysis.

To determine the proximity between cell membranes displaying PD-L1 and either PD-1 or CD8, we used the Serial Sections Alignment tool and Spatial Analysis Module in HALO. Specifically, serial sections that had been stained for the markers of interest were registered, i.e., Z-stacked, allowing for the assessment of two markers originally detected on two consecutive slides. The algorithm works by calculating the number of cells within a given distance of another cell. First, the number of CD8+ or PD-1+ cells with their cell surfaces < 20 μm from of a PD-L1+ cell was determined across the total TME (defined as the IT and PT regions combined) [16]. Then, the number of PD-L1+ cells with their cell surfaces < 20 um from a CD8+ cell or a PD-1+ cell was assessed.

### Multiplex immunofluorescence (mIF)

Tumor specimens were available for additional study with mIF from 6 patients who received anti-PD-1 [progressive disease (PD), *n* = 1; PR, *n* = 4; CR, *n* = 1]. Specimens were stained by mIF for CD8, PD-1, PD-L1, CD68, FoxP3 and neuron-specific enolase (NSE, tumor cells) as previously described [11]. In addition, 16 archival MCC tumor specimens from patients who did not receive anti-PD-1 therapy were stained by mIF for PD-1, CD8, CD4, CD20, Fox-P3, and NSE.

#### Panel 1: (PD-L1, PD-1, NSE, CD68, CD8, DAPI)

4 μm-thick slides from FFPE tissue were heated at 57 °C overnight, and the residual paraffin was removed using xylene. After tissue rehydration using a series of graded alcohols to distilled water, antigen retrieval was performed using Tris-EDTA buffer and microwave treatment. Slides were washed, and blocking was performed with 3% H2O2 blocking solution followed by Dako antibody diluent. The first primary antibody (“Position 1” in Table 1) was then applied. Opal polymer HRP Ms. + Rb (Perkin Elmer, Hopkington, MA) was used as the secondary antibody. The slides were washed, and the tyramide signal amplification (TSA)-dye (Opal 7 color kit, Perkin Elmer, Hopkington, MA) for Position 1 was applied. Slides were then microwaved to strip the primary and secondary antibodies, washed, and blocked again using blocking solution. The second primary antibody (“Position 2”) was applied, and the process was repeated through amplification of the sixth primary antibody labeling. After the last step of antibody striping, DAPI was applied. After unbound DAPI was washed off, slides were coverslipped using VectaShield Antifade Mounting Medium (Vector Labs, Burlingame, CA). Panel 1 was performed using a manual method for staining.

**Table 1:** Primary antibody information for Multiplex IHC/IF panels

Position	Antibody	Clone (host)/Company	Dilution	Incubation (min)	TSA dye
Panel 1					
1	PD-L1	SP142 (rabbit)/Spring Bio	1:800	60	620
2	PD-1	EPR4877(2) (rabbit)/AbCam	1:1000	30	650
3	NSE	BBS/NC/VI-H14(mouse)/Dako	1:1000	60	570
4	CD68	PGM-1(mouse)/Dako	1:500	30	540
5	CD8	4B11(mouse)/AbD	1:100	30	520
6	DAPI	Perkin Elmer Opal 7-color kit	2 drops/ml	5	NA
Panel 2					
1	FoxP3	236A/E7(mouse)/abcam	1:100	30	570
2	NSE	BBS/NC/VI-H14(mouse)/Dako	1:400	30	620
3	PD1	EPR4877(2)(rabbit)/abcam	1:500	120	650
4	CD4	EP204(rabbit)/Sigma	1:50	120	540
5	CD20	L26(mouse)/Leica	1:800	30	520
6	CD8	4B11(mouse)/AbD	1:100	30	690
7	DAPI	Perkin Elmer Opal 7-color kit	2 drops/ml	5	NA

#### Panel 2: (PD-1, NSE, CD4, CD8, CD20, FoxP3, DAPI)

An automated protocol was used for Panel 2. Slides were heated at 60 °C for 30 min then Dewax (Leica Biosystems, Buffalo Grove, IL) applied to remove any paraffin. Antigen retrieval was performed using ER2 (Leica Biosystems, Buffalo Grove, IL) at 100 °C for 40 min followed by a washing step. Non-specific staining was blocked using Blocking/Ab Diluent (Perkin Elmer, Hopkington, MA) for 5 min, then the first primary antibody was applied, Table 1**,** followed by a washing step. ImmPRESS™ HRP Anti-Mouse IgG (Vector Laboratories, Burlingame, CA) was applied for 15 min. The slides were washed, and the TSA-dye (Opal 7 color kit, Perkin Elmer, Hopkington, MA) for Position 1 was applied. Slides were then heated using ER1 (Leica Biosystems, Buffalo Grove, IL) at 95 °C for 20 min to strip the primary and secondary antibodies, washed, and blocked again using Blocking/Ab Diluent. The second primary antibody (Position 2) was applied, followed by Opal polymer HRP Ms. + Rb (Perkin Elmer, Hopkington, MA). The corresponding Opal was applied, and the antibodies stripped. The staining process was repeated for positions 3–6. After the last step of antibody striping, the slides were removed from the Bond Rx (Leica Biosystems, Buffalo Grove, IL), and DAPI was applied. After unbound DAPI was washed off, slides were coverslipped using ProLong™ Diamond Antifade Mountant (Life Technologies, Waltham, MA).

### Slide scanning and analysis for multispectral IF/IHC

Stained slides were scanned using the Vectra 3.0 Quantitative Pathology Imaging System (Perkin Elmer, Waltham, MA). Ten high-power fields (HPF) along the tumor-stroma interface enriched in immune cells (“hot-spots”) were chosen for analysis in each specimen. InForm 2.3 Image Analysis software (Perkin Elmer) was used for spectral unmixing, cell segmentation, and identification and quantification of cellular subsets. The fraction of cells in each lineage was normalized by the number of tumor cells in each analyzed field.

### Tumor Merkel cell Polyomavirus (MCPyV) status

Tumor specimens were assessed for the presence of MCPyV as previously described [7, 11].

### Statistics

Data are reported as the median and range in the text and median ± IQR for figures. Two-sided Mann–Whitney U-test was used to compare tumor-infiltrating immune cell densities between responders (R) vs. non-responders (NR); patients with PD-L1+ vs. PD-L1(−) tumors; and MCPyV+ vs. MCPyV- tumors. Chi-square test was used to compare the fraction of PD-L1+ tumors and the PD-L1 expression gradient (TC and IC) between R vs. NR, and MCPyV+ vs. MCPyV- tumors.

## Results

### Patient and specimen characteristics

Twenty-six MCC patients received anti-PD-1 therapy and had pretreatment tumor tissue available for study. As of data analysis on 08/01/2016, 17 demonstrated an objective response (CR = 5, PR = 12), 8 showed no response (1 with stable disease and 7 with PD), and one patient demonstrated a transient PR that did not meet RECIST criteria. Fourteen tumors were primary lesions and twelve were metastases. The median interval between specimen acquisition and treatment initiation was 5 months (range 7 days - 8 years), with 85% of the specimens in the cohort being taken within 2 years of treatment initiation. PD-L1 IHC was performed on 25/26 specimens, CD8 IHC on 23/26, and PD-1 IF on 16/26, depending on tissue availability. The median PD-1+ and CD8+ cell densities in patients demonstrating CR vs. PR were not significantly different, thus supporting the grouping of these patients as “Responders”. The density of PD-1+ and CD8+ cells also did not differ by whether the studied specimen was from a primary lesion or a metastasis. Six specimens had sufficient material for IF multiplexing with a panel for NSE, PD-L1, PD-1, CD68, CD8, and FoxP3 expression.

To discern which cell types in the MCC TME express PD-1, a second cohort of 16 archival surgical pathology specimens was studied. Six specimens were from primary lesions and 10 from metastases. The archival specimens were previously characterized with regard to MCPyV status, PT CD8+ density, and tumor cell PD-L1 expression, and these parameters were shown to significantly associate with each other [7]. Among 26 patients who received anti-PD-1 therapy, similar findings were observed, Additional file 1: Figure S1A.

### The density of PD-1+ cells, but not CD8+ cells or viral status, correlates with response to anti-PD-1

**Fig. 2:**
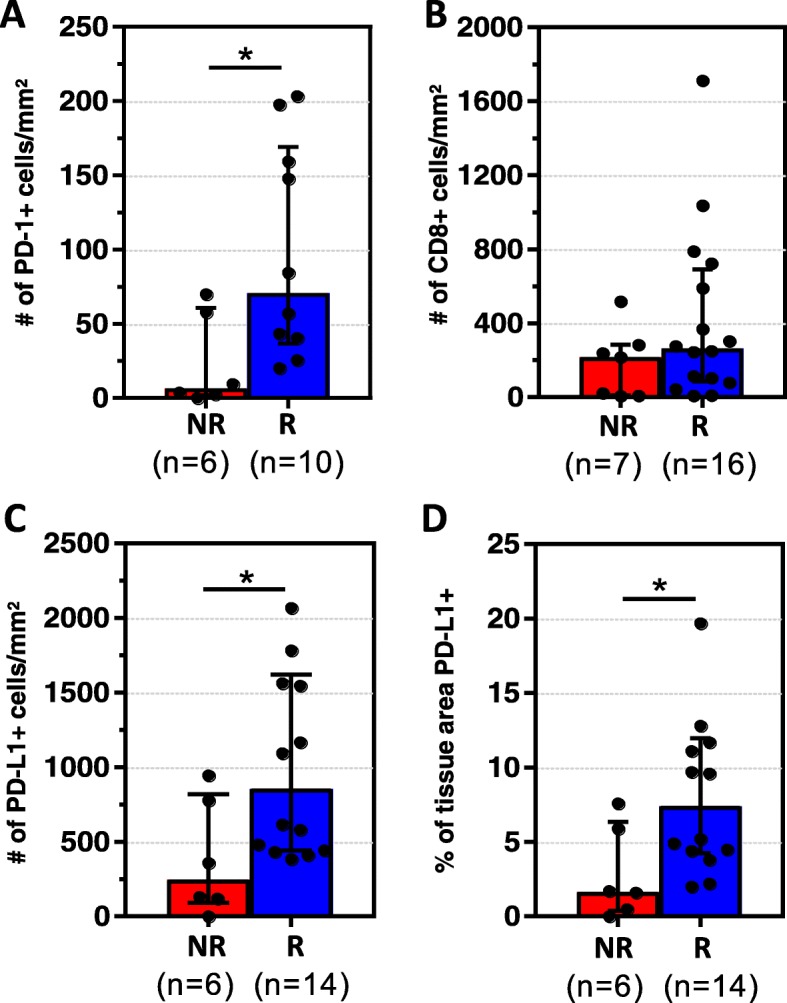
PD-1+ and PD-L1+ cell densities correlate with clinical response to anti-PD-1. **a** Responders (R) had significantly higher median densities (±IQR) of PD-1+ when compared to non-responders (NR) **b** CD8+ cell densities were not significantly different between the two groups. **c**, **d** R had higher median densities (±IQR) of PD-L1+ cells as well as PD-L1+ tissue area when compared to NR. *p < 0.05. Assessments were made on the total TME (IT+PT). Results for each individual region are presented in Additional file 1: Figures S2 and Figure S4

We evaluated the correlation between the density and distribution of PD-1+ and CD8+ cells and response to anti-PD-1. The total density of PD-1+ cells (PT + IT) was significantly higher in R vs. NR [median number of positive cells/mm^2^(range) 70.7(20.2–203.4) vs. 6.7(0–70), *p* = 0.03], Fig. 2a. In contrast, the total density of CD8+ cells did not associate with response status [R 264/mm^2^ (8.6–1712) vs. NR 216.6/mm^2^(7.0–517.0), *p* = 0.17], Fig. 2b. We further subdivided the TME into PT and IT regions and analyzed the PD-1+ and CD8+ cell densities in each area separately, and similar results were observed, Additional file 1: Figure S2. We also found that while CD8+ cell densities, especially peritumoral, associated with the presence of MCPyV, PD-1+ cell densities and response to therapy did not, Additional file 1: Figure S1b and Nghiem, et al. 2016. These findings indicate that CD8+ and PD-1+ cell densities are not interchangeable biomarkers of response to anti-PD-1 in patients with MCC.

### PD-L1 density and total PD-L1 expression area correlate with response to anti-PD-1

PD-L1 status on tumor cells (TC) + immune cells (IC), or TC or IC alone, was assigned by a pathologist as PD-L1+ or PD-L1- using a 1% threshold. An association with clinical response was not observed, Additional file 1: Figure S3A. When the percentage of all cells (TC + IC) in the TME expressing PD-L1, as well as the percentage of TC or IC, were studied at discrete intervals rather than binomially, there was still no association with response, Additional file 1: Figure S3B.

We next evaluated the correlation between digitally-quantified PD-L1 expression (a continous variable) and response to anti-PD-1. Increased densities of PD-L1+ cells (Fig. 2c**)** and an increased fraction of tissue surface area expressing PD-L1 (Fig. 2d) both correlated with improved response to PD-1 checkpoint inhibition (*p* = 0.02, *p* = 0.03, respectively). Similar associations held when the IT and PT regions were examined separately, Additional file 1: Figure S4.

To further dissect potential associations between the degree of CD8 cytotoxic T-cell infiltration and PD-1/PD-L1 expression, the cohort was divided into quartiles of PD-L1+, CD8+ and PD-1+ total cell densities. All patients in the highest quartile for each of these three markers demonstrated a response, Additional file 1: Figure S5. None of the patients with the lowest PD-1+ cell density responded to therapy, while only one patient among 5 in the lowest quartile of PD-L1 expression demonstrated a response. In contrast, several patients in the lowest quartile of CD8+ cell density showed a response. True comparison metrics of the sensitivity and specificity of these different markers will require studying larger cohorts, but these early findings suggest that PD-1+ cell density in pretreatment tumor biopsies may be a better predictor of response than PD-L1+ or CD8+ cell density.

### The density of PD-1+ lymphocytes adjacent to PD-L1+ cells correlates with clinical response to anti-PD-1 therapy

**Fig. 3:**
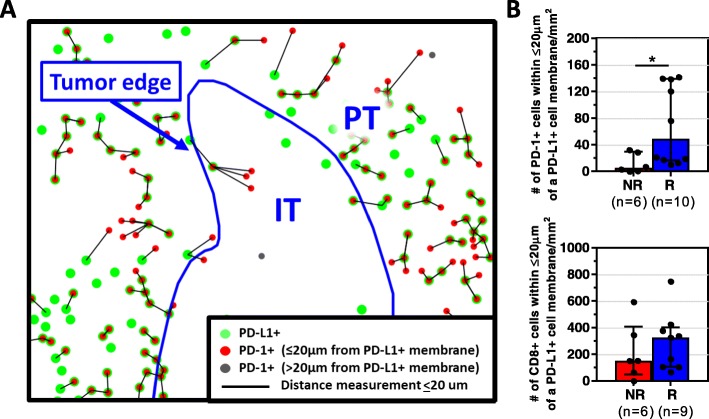
The density of PD-1+ cells adjacent to a PD-L1+ cell correlates with clinical response to anti-PD-1. **a** Representative composite image depicting proximity analysis between PD-1+ and PD-L1+ cells performed using the HALO software Spatial Analysis module, Supplemental Methods. The distance between each PD-1+ cell and the nearest PD-L1+ cell (green circles) was calculated, and only those at a distance ≤20 μm (black lines) are quantified (red circles). **b** Responders (R) had significantly higher median densities (±IQR) of PD-1+, but not CD8+, cells interacting with PD-L1+ cells compared to non-responders (NR). *p < 0.05. The density of PD-L1+ cells within  20 μm of a PD-1+ or CD8+ cell was also calculated, and a similar association with response was observed, Additional file 1: Figure S6

We have previously reported examples of constitutive PD-L1 expression in the TME, i.e.*,* not associated with an immune infiltrate [7, 17–20]. We posit that this pattern may explain why a proportion of patients with PD-L1+ tumors do not respond to anti-PD-1/PD-L1, [14, 21] as it is adaptive PD-L1 expression that indicates an endogenous antitumor immunity [22]. One way to denote adaptive (as opposed to constitutive) PD-L1 expression is the close proximity of PD-L1+ cells in the TME to TILs [17]. As such, we calculated the density of PD-1+ or CD8+ TILs proximate to a PD-L1+ cell, Fig. 3a**,** as well as the density of PD-L1+ cells proximate to a PD-1+ or CD8 + cell. The density of PD-1+ cells adjacent to a PD-L1+ cell was significantly higher in R vs. NR [69.9/mm^2^(10.5–141.8) vs. 5.15/mm^2^(0–32.4), *p* = 0.03], Fig. 3b. In contrast, the density of CD8+ cells in close proximity to a PD-L1+ cell was not correlated with clinical response to anti-PD-1 therapy [R 326.9/mm^2^(67.3–748.8) vs. NR 152/mm^2^(1–593.7), *p* = 0.46]. When the transposed metric of PD-L1+ cell density proximate to a PD-1+ or CD8 + cell was assessed for the relationship to response, similar results were observed, Additional file 1: Figure S6. We next controlled for the density of PD-1+ and PD-L1+ cells in each sample, and Responders still exhibited a significantly higher density of PD-1+ cells in proximity to PD-L1+ cells than Non-Responders, indicating that the proximity measurement reflects more than simply another representation of PD-1+ and PD-L1+ cell density.

Preliminary results have also been reported noting the association between PD-1/PD-L1 ‘interaction’ and response to anti-PD-1 therapy in patients with melanoma [23].

### Multiplex analysis demonstrates that PD-1 is expressed on multiple cell types in MCC

**Fig. 4:**
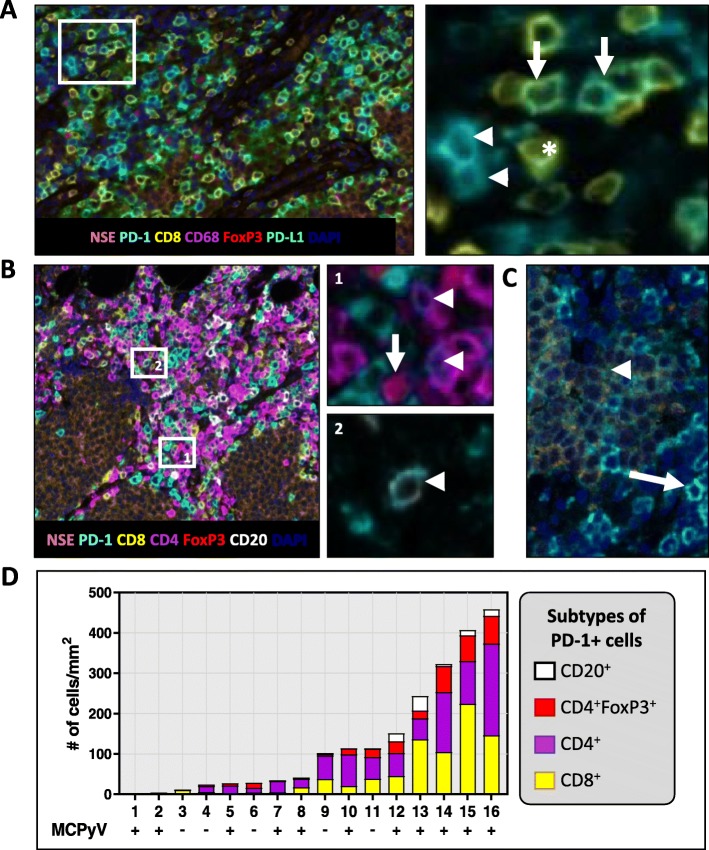
Multiplex immunofluorescence studies demonstrate that PD-1 is expressed on multiple cell types in MCC, including CD8+ cells, CD4+ cells, T_reg_ (CD4 + FoxP3+), CD20+ B-cells, and even sometimes on tumor cells. **a** Representative photomicrograph of multiplex panel (CD8, yellow; CD68, magenta; FoxP3, red; NSE (tumor), orange; PD-1, cyan; PD-L1, green and DAPI) from a responder in the cohort of patients treated with anti-PD-1. Higher magnification photomicrograph shows that while there is a significant proportion of PD-1+/CD8+ cells (arrow), there are also PD-1+ cells that are CD8- (arrowhead), and CD8+ cells that are PD-1- (asterisk). Left and right panels: 200× and 400× original magnification, respectively. **b** A second multiplex panel (PD-1, cyan; CD8, yellow; CD4, magenta; FoxP3, red; CD20, white; NSE (tumor), orange, and DAPI) was applied to archival MCC specimens to further characterize cell types expressing PD-1. Left panel: Representative photomicrograph showing host-tumor interface, 200× original magnification. Upper-right panel (1): Cell types expressing PD-1 include CD4 + FoxP3+ cells (arrow) and PD-1 + FoxP3- (arrowhead) cells, 400× original magnification. Lower-right panel (2): CD20+ B-cells (arrowhead) were also noted to express PD-1. (only CD20 and PD-1 channels are shown in the inset, 400× original magnfication). **c** In one case, low-level, constitutive PD-1 expression on nearly every tumor cell was observed (arrowhead). High levels of PD-1 expression were also seen on TIL (arrow). **d** PD-1+ cell densities across *n* = 16 tumor specimens show that PD-1 expression on multiple cell types is observed across different levels of inflammation. The virus status of each specimen is displayed below each specimen number

We performed multiplex IHC/IF on six pre-treatment specimens from patients receiving anti-PD-1 therapy to further characterize the MCC TME. We observed that while a substantial population of cells expressed both CD8 and PD-1, there were subpopulations that expressed one or the other marker, Fig. 4a. PD-1 can be expressed not only by CD8+ but also by CD4+, CD20+, T_reg_ and NK cells. As such, a second multiplex panel was designed to assess the relative proportion of PD-1 expressed by these different immune cell subsets in a cohort of archival MCC specimens, Fig. 4b. A NK marker was not included in the multiplex panel due to the very low density of NK cells in the MCC specimens shown on single IHC staining using anti-NKp46 (median density 1 cell/mm^2^).

The median density of CD8 + PD-1+ cells was 46.4/mm^2^ (range 0.1–199.4), and the median density of CD4 + PD-1+ cells (T_reg_ and T_eff_) was similar [50.5/mm^2^(0.0–278.0)]. On average, FoxP3+ cells represented approximately 22% of the CD4 + PD-1+ population. Scattered CD20 + PD-1+ cells were also found [median 5.3/mm^2^(0–30.2)]. One exceptional case demonstrated broad, constitutive PD-1 expression on tumor cells, Fig. 4c. PD-1 expression on multiple immune cell types was observed and was independent of the degree of inflammation or viral status, Fig. 4d.

## Discussion

MCC appears to be highly responsive to anti-PD-1 therapy, regardless of viral status. Although anti-PD-1 responsiveness in some other cancer types has been correlated with PD-L1 expression (“positive” or “negative”) in pretreatment tumor specimens; this has not been shown for MCC [11]. In the current study, we used sophisticated digital image analysis for cell density along with cartographic assessments and found that higher-resolution digitally-assisted quantitative measurements of the PD-1/PD-L1 axis do, in fact, associate with response to therapy. Significant factors include PD-1+ cell density, PD-L1+ cell density, total surface area within the tumor mass displaying PD-L1, and the expression of PD-1 in close proximity to a PD-L1+ cell. Importantly, we also showed a divergent result for CD8+ cells, whereby neither CD8+ cell densities nor the juxtaposition of CD8+ cells to PD-L1+ cells correlate with response in this setting.

Galon and colleagues demonstrated the prognostic utility of quantitative density assessments of lymphocyte subsets in specific geographic tumor regions for patients with colorectal carcinoma. In their seminal studies, they identified CD3+, CD8+ and CD45RO+ cells in the IT and PT regions with IHC and showed that the “Immunoscore” tiered scoring system based on cell density measurements in these areas had the power to not only augment, but sometimes surpass the predictive value of TNM staging [24, 25]. Newly available multiplexed imaging platforms have facilitated even more finely resolved spatial metrics, allowing for the enumeration of relationships between individual cells. For example, studies in pancreatic carcinoma and head and neck squamous cell carcinoma (HNSCC) have shown the association between improved prognosis and proximity of specific cell types, e.g.*,* CD8+ cells adjacent to cancer cells, and between the number of CD8+ cells next to a PD-L1+ or T_reg_ cell, respectively [26, 16]. Similar approaches were used to map the PD-L1+ microenvironmental niche for Reed-Sternberg cells in Hodgkin lymphoma [27].

In addition to assisting with prognostication, immune cell density measurements in the IT and PT regions have been studied as predictive biomarkers for response to anti-PD-1 [22, 28, 29]. The emphasis in most of the studies to date has been on CD8, rather than PD-1 expression. Our findings suggest that the precise quantification of PD-1+ cell densities could be of value to predict the response to anti-PD-1 therapy. Because PD-1 is the direct target of anti-PD-1 drugs, it stands to reason that the amount of PD-1 in the TME may be a key component of next generation biomarker panels. More specifically, anti-PD-1 agents are thought to exert their action by disrupting the PD-1/PD-L1 interface. By adding a distance assessment between these two molecules, we provide a more explicit marker of the PD-1/PD-L1 interaction. This effectively ‘corrects’ for the potential expression of one immunoactive partner too far away from a likely receptor-ligand pairing or in the absence of the other, for example, in the case of oncogene-driven or “constitutive” tumor expression.

To our knowledge, this is the first study reporting an association between PD-1+ cells densities and proximity to a PD-L1+ cell and reponse to anti-PD-1 treatment. One previous study assessed PD-1/PD-L1 distance and association with response to anti-PD-1 in patients with melanoma but reported a co-expression score (number of microscopic fields/random disks where both PD-1 and PD-L1 were expressed) [22]. Such an approach does not provide an actual distance between PD-1+ and PD-L1+ cells, and in fact, could erroneously count cells that are dual positive for PD-1 and PD-L1. In that study, the CD8 T-cells also represented the primary cellular source of PD-1 expression.

The differential association between PD-1+ and CD8+ TIL densities with response to anti-PD-1 in MCC prompted us to explore other cell types in the MCC TME expressing PD-1. We found that in addition to CD8+ cells and a singular case of constitutive tumor cell expression, PD-1 was frequently expressed on CD4+ effector cells, T_regs_, and occasional CD20+ B-cells. In fact, approximately half of the PD-1+ TILs were CD4+ (T_eff_ or T_reg_), which is consistent with studies of archival HNSCC, ovarian cancer, and Hodgkin lymphoma FFPE specimens studied by IHC/IF; [27, 30–32] and melanoma, renal cell carcinoma, and MCC specimens studied by flow cytometry [33–35]. In vitro studies show that PD-L1 engagement of PD-1 receptors on CD4+ cells causes T-cell dysfunction. CD4+ *helping* capacities (e.g., IFN-γ and TNF-α production which promote CD8+ T-cell effector programs) can be restored following administration of anti-PD-1 [36, 37]. Patients with advanced melanoma treated with pembrolizumab showed increased Ki-67 expression not only on CD8+ cells, but also CD4+ cell populations, lending in vivo support to these in vitro findings [38]. Intriguing studies suggest that antigen-specific CD4+ cells may assume cytotoxic anti-tumor capabilities following immune checkpoint blockade [39, 40]. This mechanism may be particularly relevant in patients with MCC and Hodgkin lymphoma, both of which demonstrate high response rates to PD-1/PD-L1 checkpoint blockade despite reduced MHC class I expression [41, 42]. The functional role of PD-1 on B-cells and T_regs_ is not as well studied, but recent results suggest that anti-PD-1 antibodies may also exert anti-tumor functions by arresting suppressive B-cells and T-cells, both of which express high levels of PD-1 [43, 44].

MCC is an extremely rare cancer, affecting fewer than 3000 patients each year in the US, and as such, the primary limitation of this study is the number of specimens and amount of material per specimen available for study. Our findings will need to be confirmed in larger MCC cohorts. Due to the limited FFPE material, we were not able to apply our multiplex panels on specimens from patients treated with anti-PD-1. Thus we were not able to assess relative impact of PD-1 expression on CD8+ and CD4+ cells as well as PD-L1 expression on macrophages and tumor cells as they relate to anti-PD-1 response. Lastly, it will also likely be of value to study on-treatment specimens, which have the potential to further inform mechanisms of response and resistance to anti-PD-1 in this tumor type.

## Conclusions

The complexity of the TME has surpassed the digital reads of single-stain IHC as positive vs. negative. Value is gained by quantitating the density of cells expressing PD-L1 or PD-1. The addition of spatial metrics, such as the density of PD-1+ cells within a given distance of a PD-L1+ cell, adds a new feature to predictive biomarkers. By incorporating both partners of the receptor-ligand pair, this parameter more accurately reflects the fundamental mechanism underlying PD-1/PD-L1 blockade. Lastly, evolving multiplexing technologies facilitate studies of marker co-expression. We were able to use these techniques to identify cell types beyond CD8 in the MCC TME expressing PD-1. While future studies are needed to characterize the relative contributions of each cell type participating in the anti-tumor response, our study supports the evolving concept that lymphocytic populations beyond CD8+ cytotoxic T-cells may promote tumor regression following anti-PD-1 administration.

## Additional file

Additional file 1 **Figure S1.** CD8+ cell densities correlate with the presence of McPyV, but PD-1 densities do not correlate with viral status. **Figure S2.** PD-1+ cell densities in both the peritumoral and intratumoral regions correlate with anti-PD-1 response, but CD8+ cell densities in these regions do not. **Figure S3.** Pathologist scores for PD-L1 expression levels did not associate with response to anti-PD-1 in patients with MCC. **Figure S4.** Computer-assisted quantitation of PD-L1 in the PT and IT regions of tumor can help distinguish anti-PD-1 responders (R) from non-responders (NR). **Figure S5.** CD8+, PD-1+, and PD-L1+ TME cell densities by quartile from MCC patients receiving anti-PD1. **Figure S6.** The density of PD-L1+ cells adjacent to a PD-1+ cell correlates with clinical response to anti-PD-1. (PDF 611 kb)
