# Supplementary material for: Multidimensional, quantitative assessment of PD-1/PD-L1 expression in patients with Merkel cell carcinoma and association with response to pembrolizumab
Source: J Immunother Cancer. 2018 Oct 1;6:99. doi: 10.1186/s40425-018-0404-0 (PMC6167897; doi:10.1186/s40425-018-0404-0)
Supplement: Supplementary file 1 — Figure S1. CD8+ cell densities correlate with the presence of McPyV, but PD-1 densities do not correlate with viral status. Figure S2. PD-1+ cell densities in both the peritumoral and intratumoral regions correlate with anti-PD-1 response, but CD8+ cell densities in these regions do not. Figure S3. Pathologist scores for PD-L1 expression levels did not associate with response to anti-PD-1 in patients with MCC. Figure S4. Computer-assisted quantitation of PD-L1 in the PT and IT regions of tumor can help distinguish anti-PD-1 responders (R) from non-responders (NR). Figure S5. CD8+, PD-1+, and PD-L1+ TME cell densities by quartile from MCC patients receiving anti-PD1. Figure S6. The density of PD-L1+ cells adjacent to a PD-1+ cell correlates with clinical response to anti-PD-1. (PDF 611 kb) [file 40425_2018_404_MOESM1_ESM.pdf]

Supplemental Material

Figure S1. CD8+ cell densities correlate with the presence of McPyV, but PD-1 densities do not correlate with viral status

Figure S2. PD-1+ cell densities in both the peritumoral and intratumoral regions correlate with anti-PD-1 response, but CD8+ cell densities in these regions do not.

Figure S3. Pathologist scores for PD-L1 expression levels did not associate with response to anti-PD-1 in patients with MCC.

Figure S4. Computer-assisted quantitation of PD-L1 in the PT and IT regions of tumor can help distinguish anti-PD-1 responders (R) from non-responders (NR).

Figure S5. CD8+, PD-1+, and PD-L1+ TME cell densities by quartile from MCC patients receiving anti-PD1.

Figure S6. The density of PD-L1+ cells adjacent to a PD-1+ cell correlates with clinical response to anti-PD-1.

Figure S1

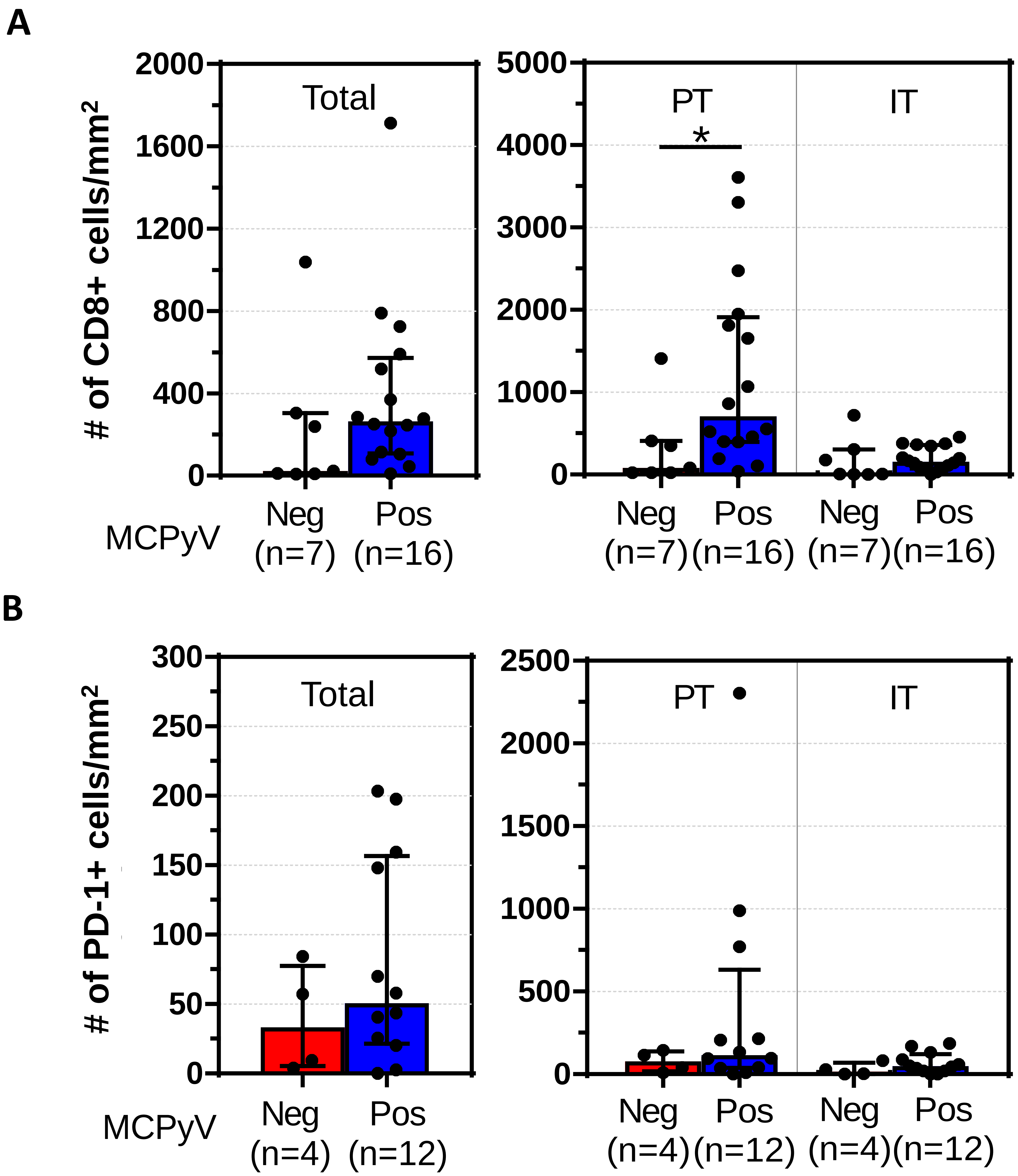

Figure S1. CD8+ cell densities correlate with the presence of McPyV, but PD-1 densities do not correlate with viral status. (A) Virus+ specimens were more likely to contain a higher density of CD8+ cells, especially in the peritumoral (PT) region as compared to MCPyV- ones (707.4 vs. 81.3 cells/mm^2, p=0.01). The virus+ specimens were also more likely to have PD-L1 expressed on $\geq$1% of tumor cells (75% vs. 30%, p=0.08, data not shown). **(B)** Neither total [PT + intratumoral (IT) regions], PT or IT PD-1+ cell density associated with viral status. *p $\leq$0.05.

Figure S2

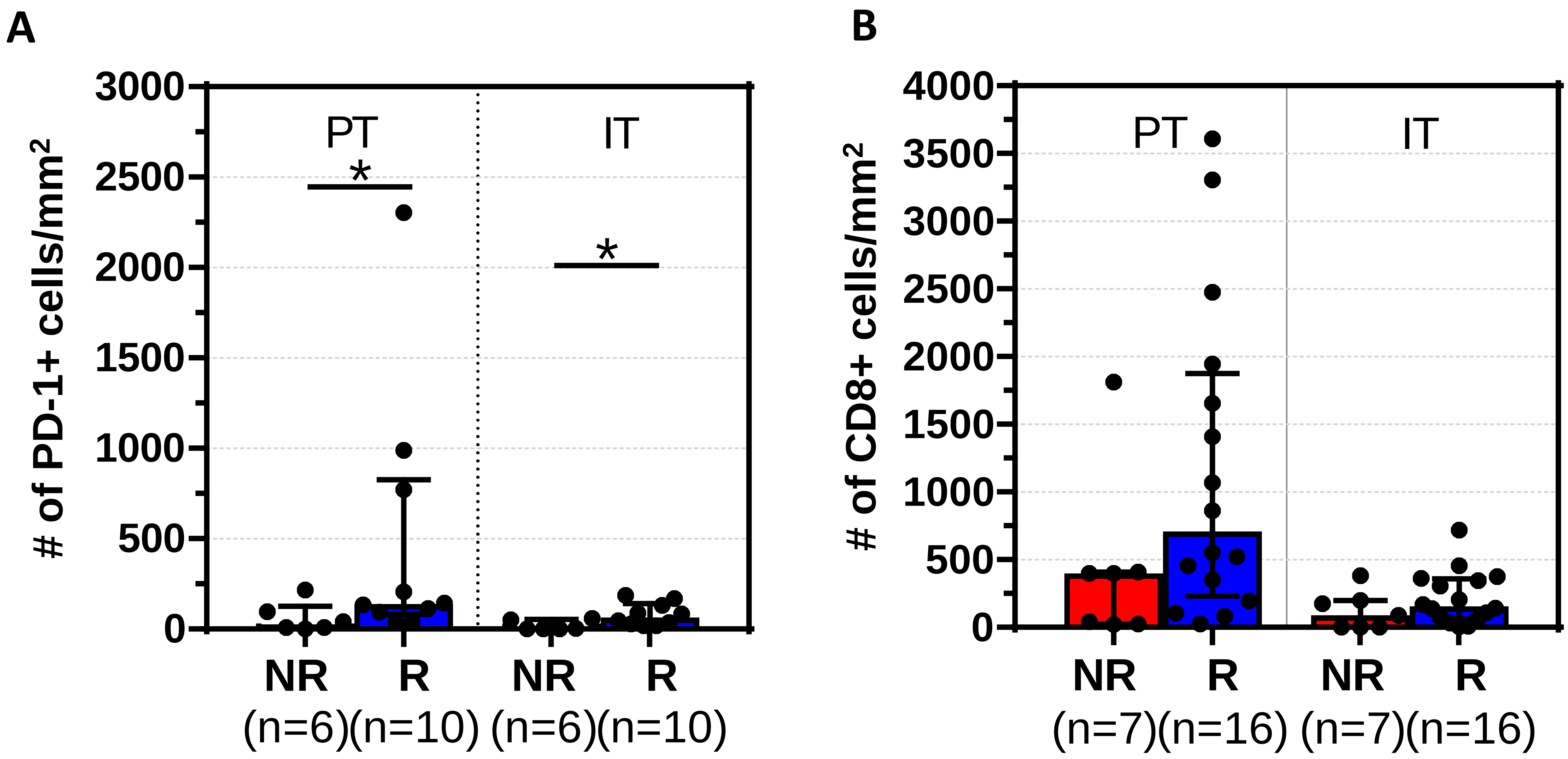

Figure S2. PD-1+ cell densities in both the peritumoral and intratumoral regions correlate with anti-PD-1 response, but CD8+ cell densities in these regions do not. (A) The density of PD-1+ cells was higher in both the PT and IT regions from responders compared to non-responders ($p=0.03$ and $p=0.05$, respectively). **(B)** Neither the CD8+ cell densities in the IT or PT region significantly correlated with response ($p=0.08$ and $p=0.22$, respectively). *p ≤ 0.05.

Figure S3

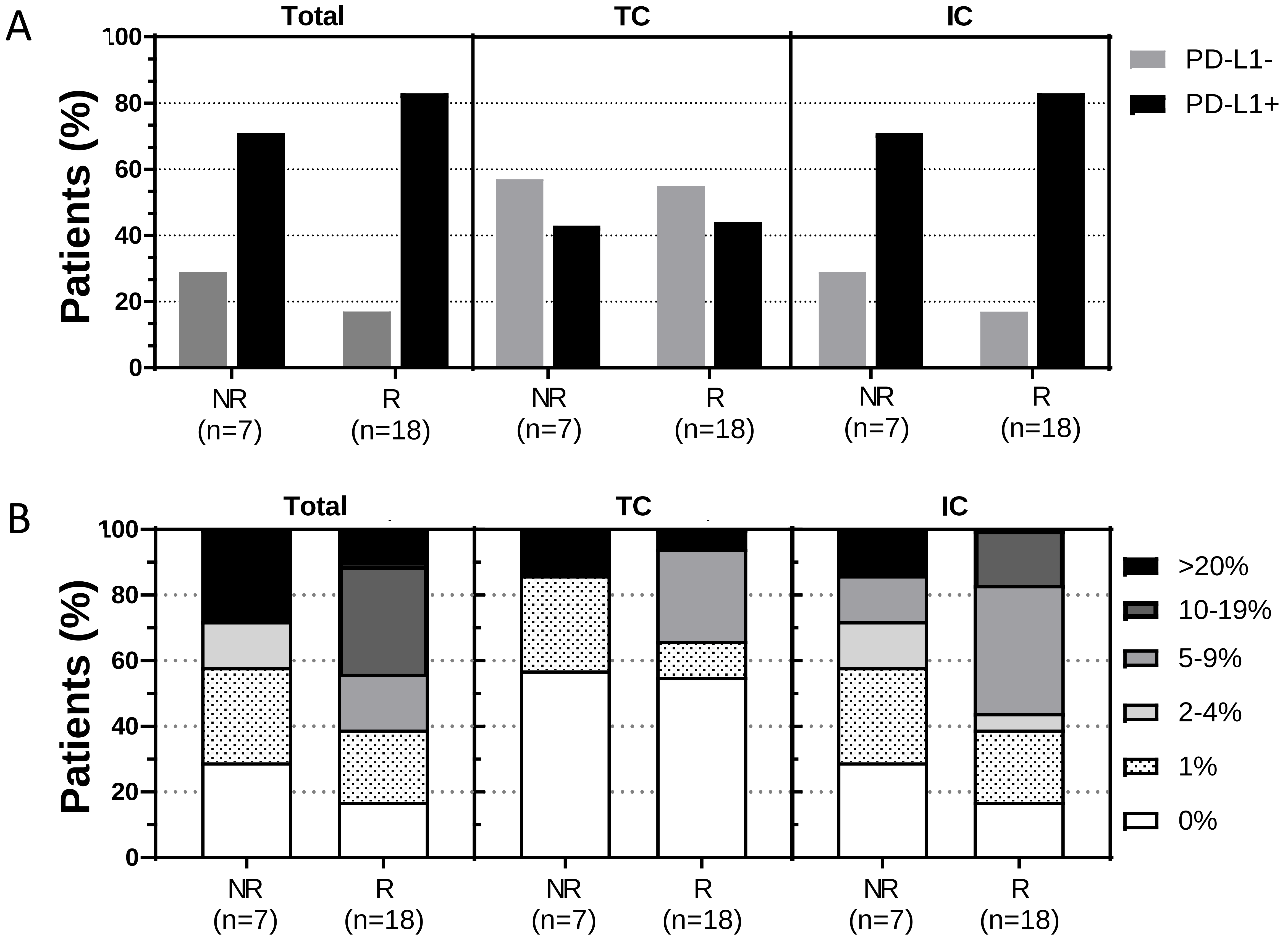

Figure S3. Pathologist scores for PD-L1 expression levels did not associate with response to anti-PD-1 in patients with MCC. (A) PD-L1 expression on both TC and IC was quantified by two pathologists. PD-L1 status (PD-L1+ specimens defined as ≥1% immune and/or tumor cells expressing PD-L1) was not associated with response to anti-PD-1. Total (TC+IC), p=0.9; TC, p=1.0; IC, p=0.6). **(B)** Increasing levels of PD-L1 expression, as scored by pathologists at discrete intervals, also were not associated with response. Total (TC+IC), p=0.18; TC, p=0.31; IC, p=0.35).

Figure S4

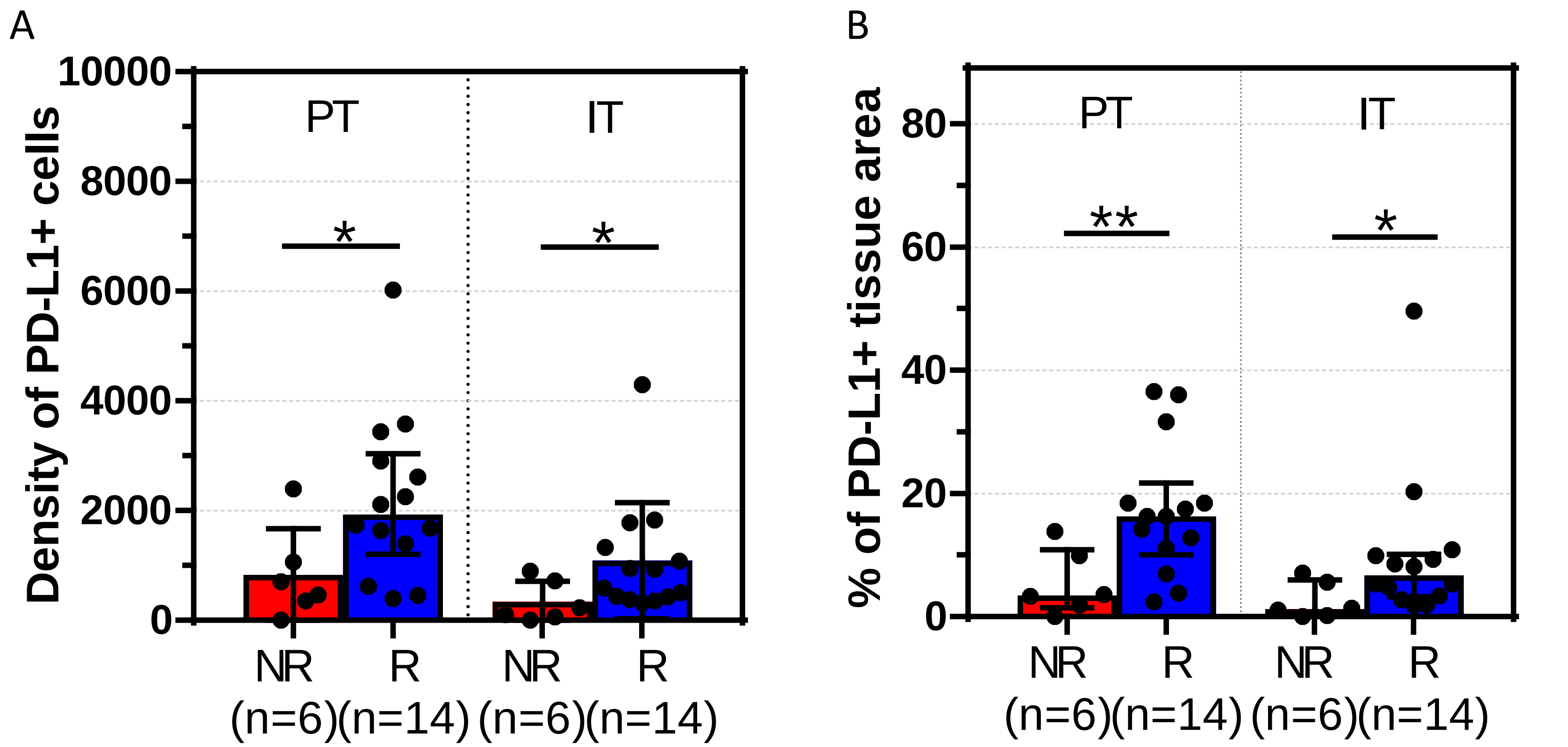

Figure S4. Computer-assisted quantitation of PD-L1 in the PT and IT regions of tumor can help distinguish anti-PD-1 responders (R) from non-responders (NR). **(A)** We also found that the density of PD-L1+ cells was higher in both the PT and IT regions from responders when compared to non-responders [median (range) 1927.0/mm^2(398.6-6017) vs. 584.7/mm^2 (0-2394.0), p=0.04; and 757.3/mm^2 (322.7-4299) vs. 166.8/mm^2 (0-894.4), p=0.02]. **(B)** When subdivided according to tissue area, responders displayed a significantly higher percentage of PT and IT PD-L1+ area (16.3%(2.4-36.5) and 6.7%(1.8-49.6), respectively) than non-responders (3.4%(0-13.8), p=0.006; and 1.2%(0-7.1), p=0.03, respectively). *p $\leq$0.05, ** p $\leq$0.01.

Figure S5

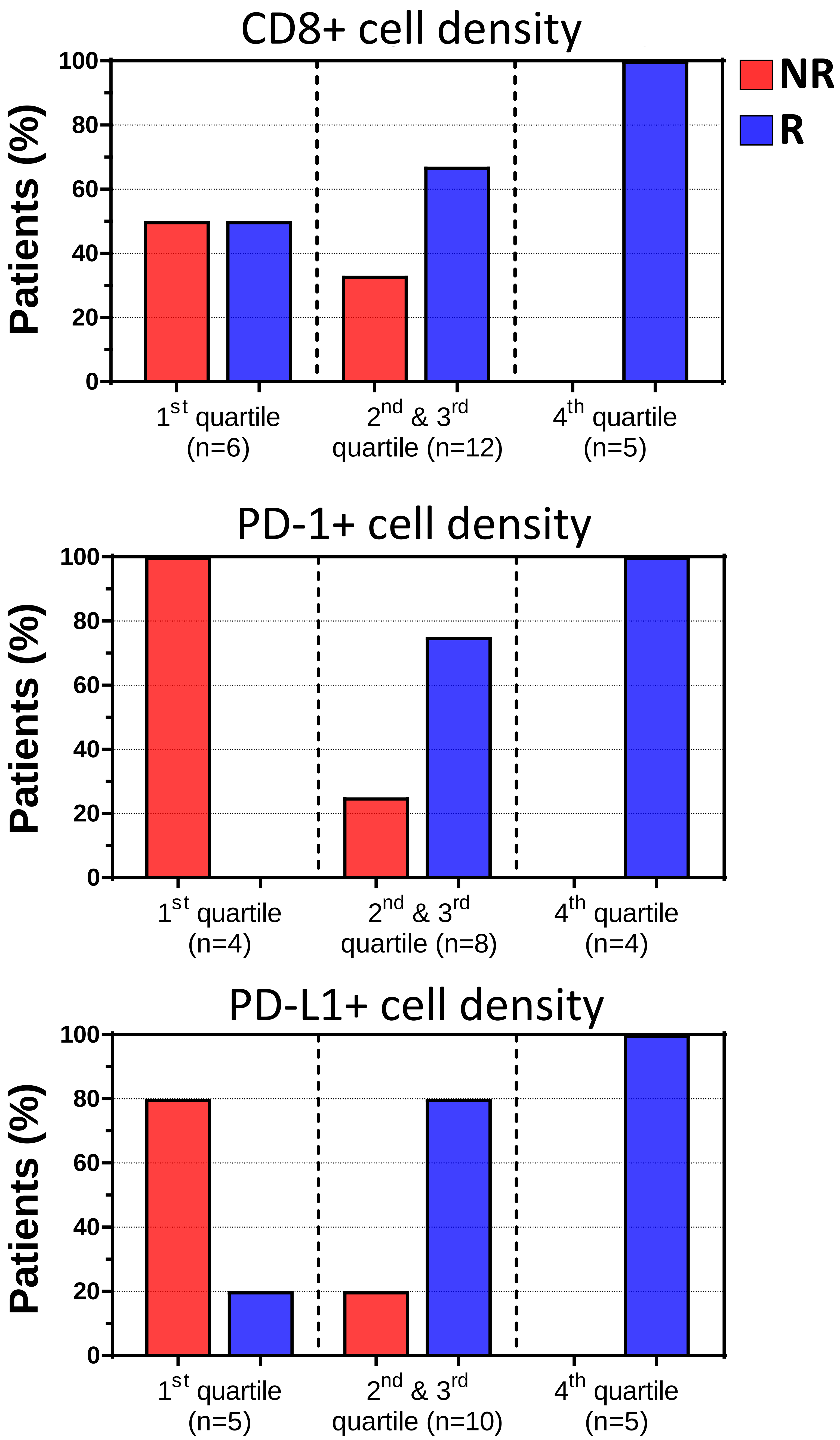

Figure S5. CD8+, PD-1+, and PD-L1+ TME cell densities by quartile from MCC patients receiving anti-PD1 .

Figure S6

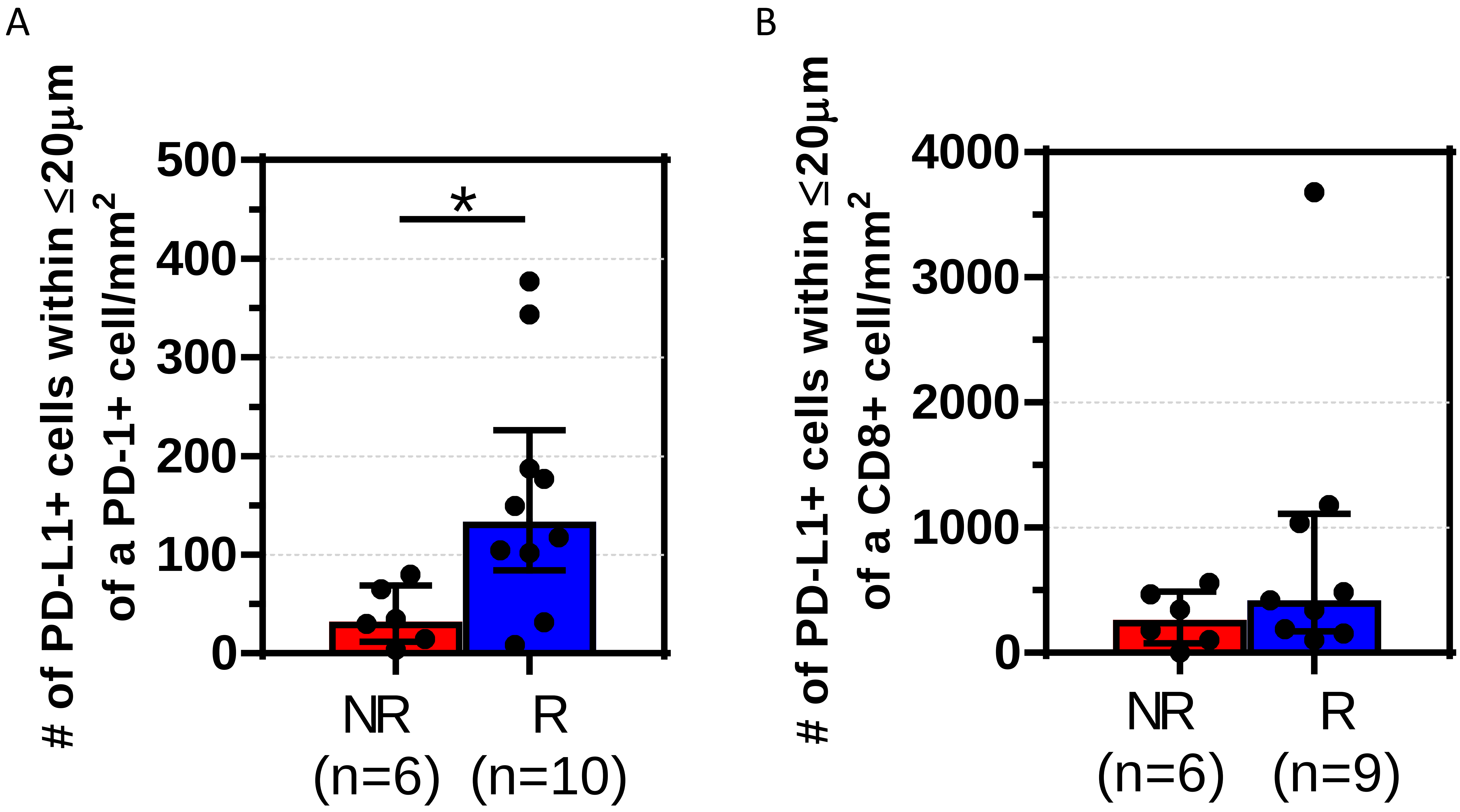

Figure S6. The density of PD-L1+ cells adjacent to a PD-1+ cell correlates with clinical response to anti-PD-1. Responders (R) had significantly higher median densities (±IQR) of PD-L1+ cells interacting with PD-1+ cells (left panel), but not CD8+ (right panet), compared to non-responders (NR). *$p \leq 0.05$.
